# The Posterior Transtriceps Approach for Intra-articular Elbow Arthrography: A Painless Method?

**DOI:** 10.7759/cureus.31642

**Published:** 2022-11-18

**Authors:** Alan Alexander, Kambiz Motamedi, Jonathan Chen

**Affiliations:** 1 Radiology, University of California Los Angeles, Los Angeles, USA

**Keywords:** radiocapitellar, transtriceps, elbow, mri, arthrogram

## Abstract

We determined the diagnostic and patient experience advantage of the transtriceps approach for elbow arthrograms.

MRIs of two patients who underwent an MRI arthrogram of the elbow in May and June 2022 were retrospectively evaluated. All procedures were performed under fluoroscopic guidance with the patient in the prone position and the arm of interest extended above the head, and the elbow of interest flexed at 90 degrees. A 25 gauge needle was utilized. All MRI examinations were performed on 3-Tesla MRI scanners (Siemens, Hamburg, Germany) and in accordance with our institution's standard elbow arthrogram MRI protocol. The arthrogram was deemed successful if contrast was visualized in the elbow joint, and the MRI examinations were assessed for diagnostic ability and extra-articular leakage. Patient comfort and pain were also assessed.

Both arthrograms demonstrated adequate contrast in the elbow joint, and the MRIs confirmed no leakage of contrast or bubbles. The patients felt 0 pain during the procedure on a scale of 0-10.

Although the radiocapitellar approach is commonly used for elbow arthrograms, we found the transtriceps approach to be diagnostically sound, painless, and easier to perform.

## Introduction

In 2009, Lohman et al. discussed the transtriceps approach for elbow arthrography and found that the major advantage was that if contrast leakage were to occur, it wouldn’t interfere with the diagnostic quality given the leakage wouldn’t interfere with the lateral structures, as would the case with a more conventional and commonly used radiocapitellar approach [1]. In 2012, Wagenberg et al. reported studying a larger patient cohort using the transtriceps approach and found there is less contrast leakage compared to the radiocapitellar approach [2]. Furthermore, the transtriceps approach avoids damage to the radial collateral ligament [1].

Most published arthrogram guides suggest and describe the radiocapitellar approach [3,4]. Our institution historically uses the radiocapitellar approach, though we decided to evaluate the transtriceps approach given the aforementioned reasons and to assess if it is a technically easier technique and causes the patient less discomfort.

## Technical report

Two elbow MRI arthrograms using a transtriceps approach were performed in May and June of 2022. One patient was male and one was female, both between 18 and 29 years of age.

**Figure 1:**
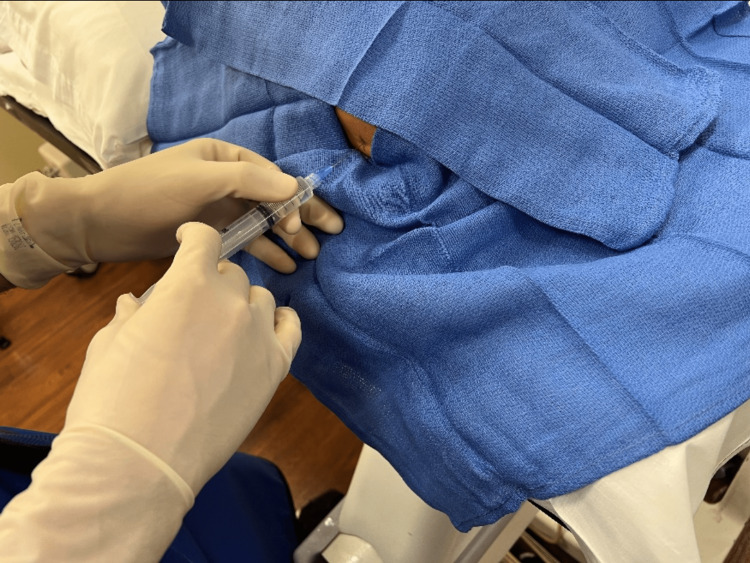
Injection Needle advancement into the posterior fat pad whilst injecting 1% lidocaine.

**Figure 2:**
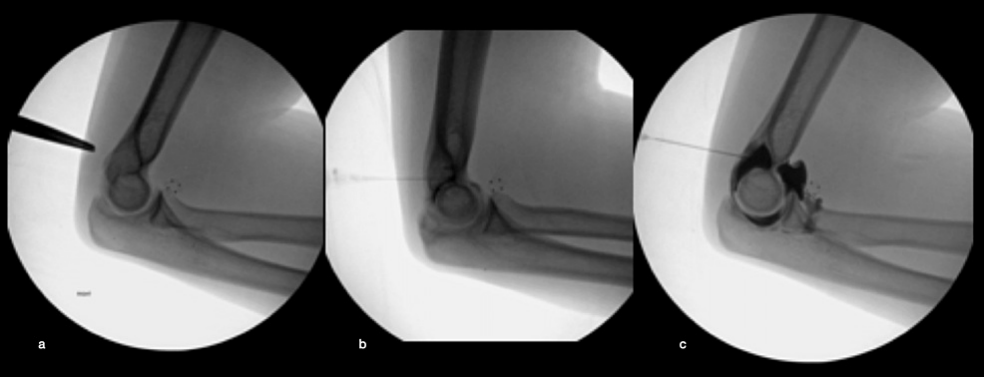
Fluoroscopic Needle Entry and Injection (a) Marking the entry point overlying the posterior fat pad in line with the deepest portion of the olecranon fossa. (b) After the needle tip is placed in the deepest aspect of the olecranon fossa, a small amount of iodinated contrast is injected demonstrating opacification of the joint and confirming appropriate positioning. (c) Diluted gadolinium (1:200) is administered into the elbow joint, demonstrating distention and opacification of the joint capsule.

The elbow arthrogram was performed by lying the patient prone on a standard fluoroscopy table and placing their affected arm extended above their head. The elbow was flexed to 90 degrees and the patients rested their arm on the table. The posterior fat pad superior to the olecranon was palpated and under fluoroscopic guidance, the entry site was marked to be in line with the deepest aspect of the olecranon fossa (Figure 1). The patient’s elbow was then prepped and draped using a sterile technique. Using a 25 gauge 1.5cm needle, 4-5cc of 1% lidocaine was administered at the entry site and during needle advancement into the olecranon fossa (Figure 2a). Once the needle was deemed in the appropriate position (with the tip in the deepest aspect of the olecranon fossa), the syringe was removed and the needle was left in place. A new syringe with iodinated contrast was attached to the needle and less than 1cc of contrast was administered to confirm placement within the joint by visualizing joint opacification (Figure 2b). The iodinated syringe was removed and a syringe with 1:200 gadolinium:saline was attached. Around 6-7cc of the diluted gadolinium contrast was administered (Figure 2c). The syringe was removed, then the needle was removed, and the entry site was cleaned with peroxide. A sterile bandage was placed at the entry site, the patient’s pain was assessed, and the patient was escorted to the MRI suite.

MRIs were performed at our institution on Siemens 3-Tesla devices (Siemens, Hamburg, Germany). Studies were performed in accordance with our institution’s standard elbow MRI arthrogram protocol which included T1 with fat saturation in three planes (coronal, sagittal, axial), coronal T2 with fat saturation, and coronal thin-cut gradient echo (double-echo-steady-state (DESS)).

**Figure 3:**
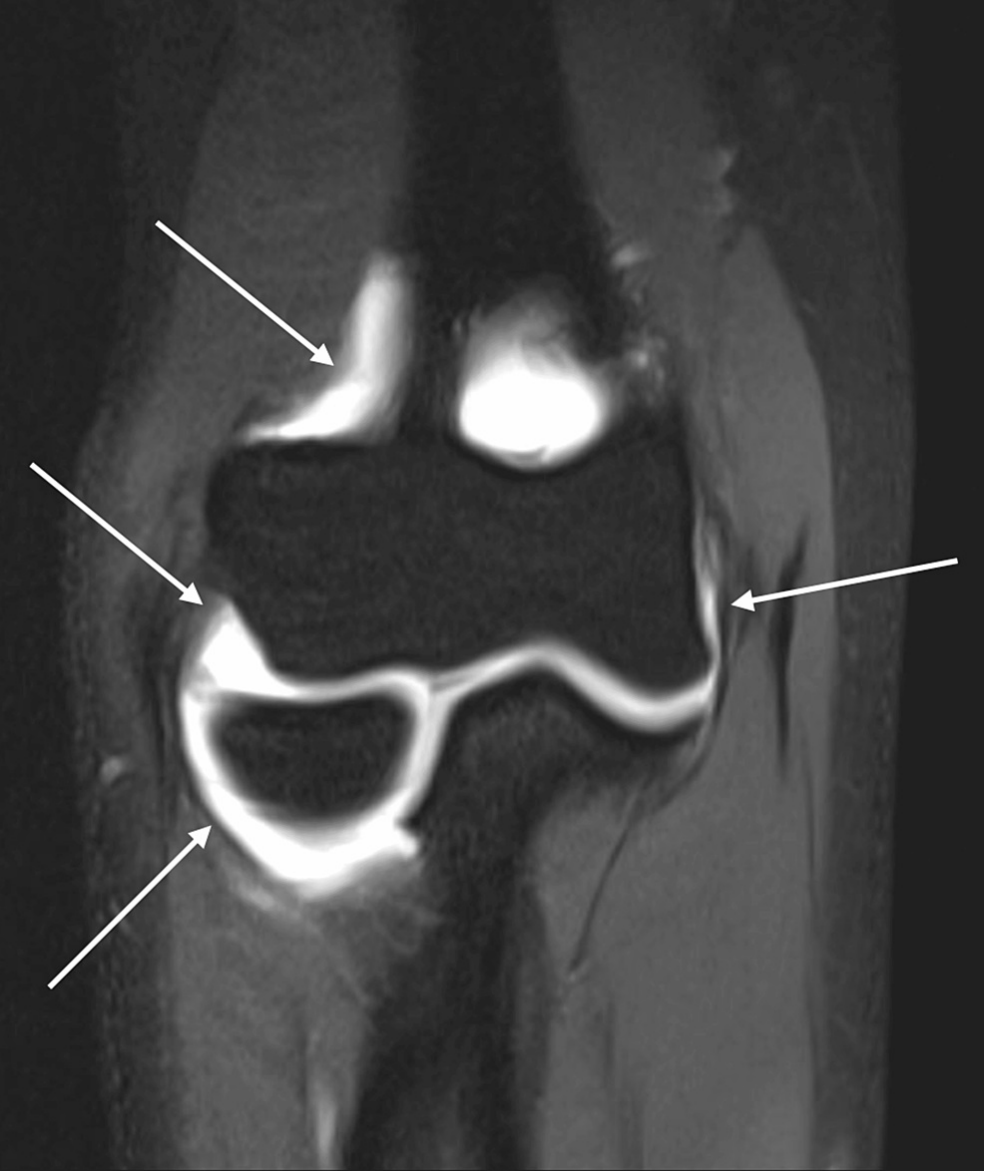
MRI Arthrogram MRI post arthrogram demonstrating adequate distention of the elbow joint capsule (arrows) without extravasation.

Both patients endorsed 0 pain on a scale of 0-10 during and after the procedure. Both MRIs were of diagnostic quality with appropriate distention of the joint capsule and without contrast leakage or air bubbles (Figure 3).

## Discussion

We found the transtriceps approach to be compelling. The fat pad was easy to palpate, and the needle was easily advanced in between the humeral condyles into the olecranon fossa in one attempt for each patient. Given we are a teaching institution with fellows and residents, we feel that this approach is easier to perform and has less complications, even for those who do not demonstrate legerdemain. There was no contrast leakage in either of our two patients. If there was leakage, as in the cases previously reported by other authors [1,2], it would not affect the diagnostic quality of the MRI examination, unlike with a radiocapitellar approach. Additionally, another advantage of the transtriceps approach is that it can be transposed to ultrasound-guided arthrography [1].

Furthermore, neither of our patients felt any pain or discomfort (0/10 for both) during the procedure. One patient had a prior MRI arthrogram on the same elbow in the prior year at our institution, using a radiocapitellar approach, and stated he experienced 5/10 pain during that procedure. This could potentially be explained by the sensory nervous system thought to typically be of little importance to adipose tissue, and the transtriceps approach is entering the joint through mostly fat, whereas the radiocapitellar approach traverses more muscle and ligament structures [5,6].

## Conclusions

Although our study is limited in power, our early findings are very promising. We enthusiastically re-introduce a transtriceps approach due to the ease of procedure, decreased risk of damage to the lateral ligaments, and decreased leakage risk (and if there is leakage, it will less likely affect the diagnostic quality). Furthermore, what we found to be one of the most compelling factors for the transtriceps approach was patient comfort and lack of patient pain.
